# Identification and Validation of Prognostically Relevant Gene Signature in Melanoma

**DOI:** 10.1155/2020/5323614

**Published:** 2020-05-08

**Authors:** Yali Gao, Yaling Li, Xueli Niu, Yutong Wu, Xiuhao Guan, Yuxiao Hong, Hongduo Chen, Bing Song

**Affiliations:** ^1^Department of Dermatology, The First Hospital of China Medical University, 110001 Shenyang, China; ^2^NHC Key Laboratory of Immunodermatology, China Medical University, 110001 Shenyang, China; ^3^Key Laboratory of Immunodermatology, Ministry of Education, 110001 Shenyang, China; ^4^School of Dentistry, Cardiff University, Heath Park, Cardiff CF14 4XY, UK

## Abstract

**Background:**

Currently, effective genetic markers are limited to predict the clinical outcome of melanoma. High-throughput multiomics sequencing data have provided a valuable approach for the identification of genes associated with cancer prognosis.

**Method:**

The multidimensional data of melanoma patients, including clinical, genomic, and transcriptomic data, were obtained from The Cancer Genome Atlas (TCGA). These samples were then randomly divided into two groups, one for training dataset and the other for validation dataset. In order to select reliable biomarkers, we screened prognosis-related genes, copy number variation genes, and SNP variation genes and integrated these genes to further select features using random forests in the training dataset. We screened for robust biomarkers and established a gene-related prognostic model. Finally, we verified the selected biomarkers in the test sets (GSE19234 and GSE65904) and on clinical samples extracted from melanoma patients using qRT-PCR and immunohistochemistry analysis.

**Results:**

We obtained 1569 prognostic-related genes and 1101 copy-amplification, 1093 copy-deletions, and 92 significant mutations in genomic variants. These genomic variant genes were closely related to the development of tumors and genes that integrate genomic variation. A total of 141 candidate genes were obtained from prognosis-related genes. Six characteristic genes (*IQCE*, *RFX6*, *GPAA1*, *BAHCC1*, *CLEC2B*, and *AGAP2*) were selected by random forest feature selection, many of which have been reported to be associated with tumor progression. Cox regression analysis was used to establish a 6-gene signature. Experimental verification with qRT-PCR and immunohistochemical staining proved that these selected genes were indeed expressed at a significantly higher level compared with the normal tissues. This signature comprised an independent prognostic factor for melanoma patients.

**Conclusions:**

We constructed a 6-gene signature (*IQCE*, *RFX6*, *GPAA1*, *BAHCC1*, *CLEC2B*, and *AGAP2*) as a novel prognostic marker for predicting the survival of melanoma patients.

## 1. Introduction

Among all newly diagnosed primary malignancies worldwide (excluding nonmelanoma skin cancer), 232,100 (1.7%) of which are cutaneous melanoma cases [1]. Skin melanoma causes approximately 55,500 annual deaths globally, accounting for 0.7% of all cancer deaths, which ultimately results from the metastasis of melanoma [2]. Metastatic melanoma in the small intestine is common, as the skin melanoma tends to metastasize to the gastrointestinal tract [3]. The morbidity and mortality of this disease vary according to the time of detection and accessibility to treatment. Melanoma can be roughly divided into chronic sun-damaged (CSD) or nonchronic sun-damaged (non-CSD) melanoma, which refers to long-term exposure to sunlight or non-long-term damage, respectively. CSD melanoma is usually observed in the elderly (>55 years old) and located on the posterior area of the distal head/neck region. This condition is associated with neurofibrin (*NF1*), *NRAS*, *BRAF*, non-*V600E*, or *KIT*-associated mutations, which shows high rates of mutation, while non-CSD melanoma usually affects areas of the body that are more frequently exposed to intermittent sunlight (e.g., the torso), and it is observed in younger individuals (<55 years old) who do not show significant solar elastic tissue disease. Non-CSD melanoma is associated with moderate mutation burdens, including *BRAF V600E* mutations [4]. Various approaches have been applied in the clinical treatment of melanoma, including surgery, targeting agents, and immunotherapy [5]. Even though significant advances in these treatments have been made, there are still more than 95% of patients with melanoma metastases die within one year [6]. Therefore, there exists an urgent need to identify prognostic biomarkers which can aid clinicians to accurately predict clinical outcome of melanoma and provide a reference for personalized medicine.

In the past few decades, a number of genetic or epigenetic changes have been reported to be associated with the development and progression of melanoma. Multiple driver mutations, such as *CDKN2A*, *BRAF*, *RAS*, *GNAQ*, *PTEN*, and *TP53* have also been related to the occurrence of melanoma [7]. Mutations in *RAS* can lead to activation of the receptor tyrosine kinase-*MAPK* pathway in cancer development and *BRAF* dysregulation which occurs in melanoma progression and shows a strong correlation with melanoma metastasis [8].

A number of studies have been directed towards identifying predictive survival biomarkers and establishing guidelines for the long-term prognosis of melanoma. These potential markers can mainly be divided into two categories: (1) individual molecules as independent prognostic indicators such as MCAM/MUC18 and/or other novel markers currently under study and (2) analyses of high-throughput gene expression profiles, involving several to dozens of prognostic genes for construction of gene signature [9, 10]. There exist several biological methods that can be utilized to identify gene biomarkers associated with melanoma prognosis and construct gene features [11–13]. However, the prognosis, diagnosis, and treatment strategies of melanoma still need improving. Accordingly, the purpose of this study is to analyze biological functions of bioinformatics to identify gene signals associated with the prognosis of melanoma. Altogether, our findings will provide new prognostic biomarkers of melanoma.

In order to effectively identify a reliable melanoma prognosis-related gene signature, we obtained the large dataset from the TCGA and GEO databases of melanoma patients. Gene expression profiling, single nucleotide mutations, copy number variation data, and screening of prognostic markers by integrating genomics and transcriptomics data were used to create a 6-gene signature. Verification of survival predictions was achieved through internal test sets and external validation sets. We found that this 6-gene signature was involved with important biological processes and pathways in melanoma. Similar results were obtained from GSEA analysis, suggesting that this 6-gene signature can effectively predict the prognosis risk of melanoma and provide a basis for a better understanding of the molecular mechanism of melanoma. In addition, the findings can improve the rational use of precise medications for melanoma.

## 2. Materials and Methods

### 2.1. Data Download and Preprocessing

TCGA RNA-Seq data from the UCSC cancer browser (https://xenabrowser.net/datapages/), clinical follow-up information, and copy number variation data for the SNP 6.0 chip were downloaded. A mutation comment file (MAF) was downloaded from the GDC client. GSE19234 and GES65094 expression profile data and clinical follow-up information were downloaded from the GEO database and processed them using the R package “GEOquery” to further standardize the data through scale. Initially, the RNA-Seq FPKM data from TCGA were downloaded. We selected half of the samples as the training set and the remainder as the test set. The random seed was set: seed (0). Both the training and the test sets were processed and using the R package “DESeq2” to further standardize the data through scale. The TCGA training set contained 231 samples, the test set contained 231 samples, the GSE19234 contained 44 samples, and the GSE65904 contained 214 samples (we excluded 4 samples out of 214 for missing information of survival). We collected four datasets on the specific distribution of patient age, survival status, gender, T stage, N stage, M stage, and tumor stage.  Table 1 shows the demographic and clinical characteristics of the training and validation sets. The result was analyzed by Student's *t*-test or chi-square test.

### 2.2. Multigroup Data Preliminary Analysis to Obtain Prognosis Genes

For TCGA training set samples, a univariate Cox regression analysis was used to establish the relationship between overall survival (OS) and gene expression. In this part, we identified 1569 univariate Cox regression genes with *p* value less than 0.01 as the candidate prognosis genes.

### 2.3. Copy Number Variation Data and Mutation Data Analysis

For copy number variation data in TCGA, GISTIC 2.0 was used to identify genes with significant amplification or deletion, as based on parameter thresholds for fragments with amplification or deletion lengths greater than 0.1 and *p* < 0.05. Mutsig2 was used to identify genes with significant mutations, with the threshold required to be *p* < 0.05. This analysis resulted in a total of 92 genes with significant mutation frequencies. For amplified and deleted genes recognized by TCGA copy number variation, as well as mutated gene integration, we identified a total of 2286 genes involved in biological processes and pathways.

### 2.4. Determination of Best lncRNA Characteristics

**Figure 1:**
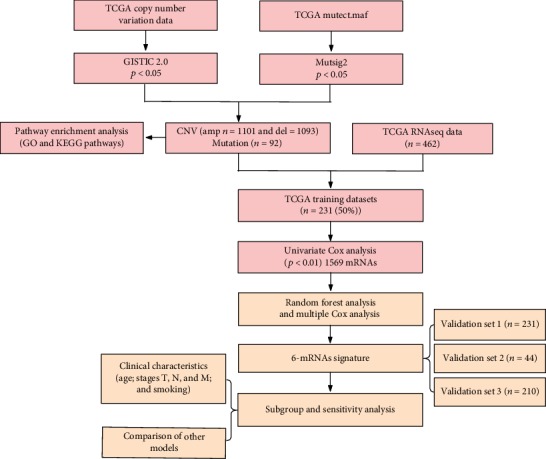
Analysis of flowchart. The flowchart indicates the exploration process and potential mechanism of melanoma prognostic genes.

To identify a gene signature, we integrated 2286 genes with copy number amplification, deletion, and mutation and 1569 prognosis-related genes and then selected the intersection of the two groups as candidate genes, which yielded 141 genes (Figure 1). The random survival forest algorithm was used to rank order prognosis genes (R package random survival forest). The parameters used were *n*_rep_ = 100, *n*_step_ = 5, representing the number of Monte Carlo iterations of 100, and the number of previous progressions was 5 [14]. Genes with relative importance greater than 0.6 were used as the final signature.

### 2.5. Establishing a 6-Gene Signature and Division of Samples in the TCGA Training Set

A multivariate Cox regression analysis method was used to establish a 6-gene signature. The model used was

(1) $$\begin{array}{c} {\text{Risk}}_{6}=0.2055267*IQCE+0.244203*RFX6+0.1858435*GPAA1+0.2002694*BAHCC1-0.329238*CLEC2B-0.08811327*AGAP2. \end{array}$$

The scoring formula for each sample was the sum of the above gene expression values∗coefficients. We then selected a sample scoring median of -0.03765742 as a cutoff and divided the samples into high-risk group and low-risk group.

### 2.6. Validation Using TCGA Test Sets and Independent Sets

In order to determine the robustness of the model, we used the same model and the same cutoff as that used in the TCGA training set and validated these results in the TCGA test set and external independent dataset GSE19234 and GSE65904. Further, we assessed the robustness of the model in all samples of the training set and validation set. The same model and cutoff of the TCGA training set were used to verify these findings in all TCGA datasets.

### 2.7. Analysis of the Clinical Independence of the 6-Gene Signature Model

To identify the independence of the 6-gene signature model in clinical applications, we used the TCGA training set, the TCGA test set, and clinical information contained within the GSE 19234 and GSE65904 data. A univariate and multivariate Cox regression was used to analyze the relevant HR, 95% CI of HR, and *p* value. We systematically analyzed the clinical information of TCGA, GSE19234, and GSE65904 patient records, including age, gender, pathology T phase, N phase, M phase, tumor stage, and 6-gene signature grouping information.

### 2.8. Use of GSEA to Analyze Pathways Enriched in High-Risk Group and Low-Risk Group

GSEA was used to determine significantly enriched pathways in the high-risk group and low-risk group of the TCGA training set. The selected gene set involved c2.cp. v6.2. symbols which contained the KEGG, BIOCARTA, and REACTOME pathways. The GSEA input file contained the expression spectrum data normalized by the TCGA training set and the sample label of the 6-gene signature. The sample label was used to mark the sample as high-risk or low-risk group. The threshold for enriched path selection was FDR *q* < 0.01, which then enabled the identification of significantly enriched paths as summarized in Table S4. Figure 1 is a flowchart of the model construction process.

### 2.9. Tissue Samples

The tumor and adjacent normal tissues of melanoma were collected from 10 patients (all participants were older than 16 years), immediately placed in liquid nitrogen, and preserved at -80°C. None of the melanoma patients received preoperative antitumor therapies. Patients and their families in this study have been fully informed, and the informed consents were obtained from the participants. This study was approved by the local Ethics Committee of Shanghai Tongren Hospital.

### 2.10. qRT-PCR

RNA extraction from cell lines and tissues was performed using TRIzol reagent (Invitrogen, Carlsbad, CA, USA). RNA was reverse-transcribed into cDNA with the QuantiTect Reverse Transcription Kit (QIAGEN, Valencia, CA, USA). Real-time PCR analyses were quantified by SYBR-Green (Takara, Otsu, Shiga, Japan), and the levels were normalized to the level of GAPDH. The sequences of the upstream and downstream primers are as follows:

*IQCE*: 5′-CGGCACTCCTGACTGTCTG-3′ and 5′-CCAGGGACATGACCGTTGC-3′. *RFX6*: 5′-AAGCAGCGGATCAATACCTGT-3′ and 5′-ACCGTGGTAAGCAAACTCCTT-3′. *GPAA1*: 5′-ACGGACGATGCGGTCAGTA-3′ and 5′-GATGCCGTACACGTTGGT-3′. *BAHCC1*: 5′-GTACCCCAGATTTTCGGGGAG-3′ and 5′-GGGTTCCATAGAAACGGTGCT-3′. *CLEC2B*: 5′-GTTCCACTCAACATGCCGAC-3′ and 5′-TGCCATCTTCAGTCCAATCCA-3′. *AGAP2*: 5′-GCAGCTACTATGAGACTTGTGC-3′ and 5′-GTGACCAACATTCGGTGAGGA-3′.

### 2.11. Immunohistochemistry

Each group of melanoma samples was fixed in 10% formalin, embedded in paraffin, and processed as 5 *μ*m continuous sections. Samples were dewaxed with discontinuous concentrations of ethanol and blocked to inhibit endogenous peroxidase. They were then heated in a microwave to retrieve antigens, cooled to room temperature, and blocked by incubation in goat serum for 30 minutes at 37°C. Samples were incubated in rabbit anti-*IQC*E, anti-*RFX6*, anti-*GPAA1*, anti-*BAHCC1*, anti-*CLEC2B*, and anti-*AGAP2* (Abcam, Cambridge, UK; 1 : 1, 200) overnight at 4°C, followed by incubation with horseradish peroxidase-coupled goat anti-rabbit secondary antibody at 37°C for 30 minutes, and stained by 3,3′-diaminobenzidine. The cell nucleus was stained blue by hematoxylin. Sections were then dehydrated, cleared by xylene, and mounted. *IQCE*, *RFX6*, *GPAA1*, *BAHCC1*, *CLEC2B*, and *AGAP2* expression was detected by immunohistochemistry using a streptavidin peroxidase method. *IQCE*, *RFX6*, *GPAA1*, *BAHCC1*, *CLEC2B*, and *AGAP2* expression in liver was taken as positive control. Samples incubated with PBS instead of *IQCE*, *RFX6*, *GPAA1*, *BAHCC1*, *CLEC2B*, and *AGAP2* primary antibody were used as negative control. Positive and negative control groups were included for each batch of immunohistochemically stained sections. The experimental procedure was performed by following the manufacturer's instructions strictly.

## 3. Results

### 3.1. Selection of Differentially Expressed lncRNA

The clinical data from the four datasets (the training set in TCGA: 231 samples, the test set in TCGA: 231 samples, the GSE19234 set: 44 samples, and the GSE65904 set: 210 samples) are summarized in Table 1. Following the analysis of the relationship between the patient's overall survival (OS) and gene expression by univariate Cox proportional hazard regression analysis, 1569 univariate Cox regression genes with a *p* value less than 0.01 were identified. Specific information regarding these candidate prognostic genes, HR of the 1569 candidate prognostic genes, coefficients, *z*-score, and *p* value used for determining the prognostic information on the upper 20 genes according to their *p* value is shown in Table 2 with detailed results presented in Table S1.

### 3.2. Copy Number Variation Data Analysis

**Figure 2:**
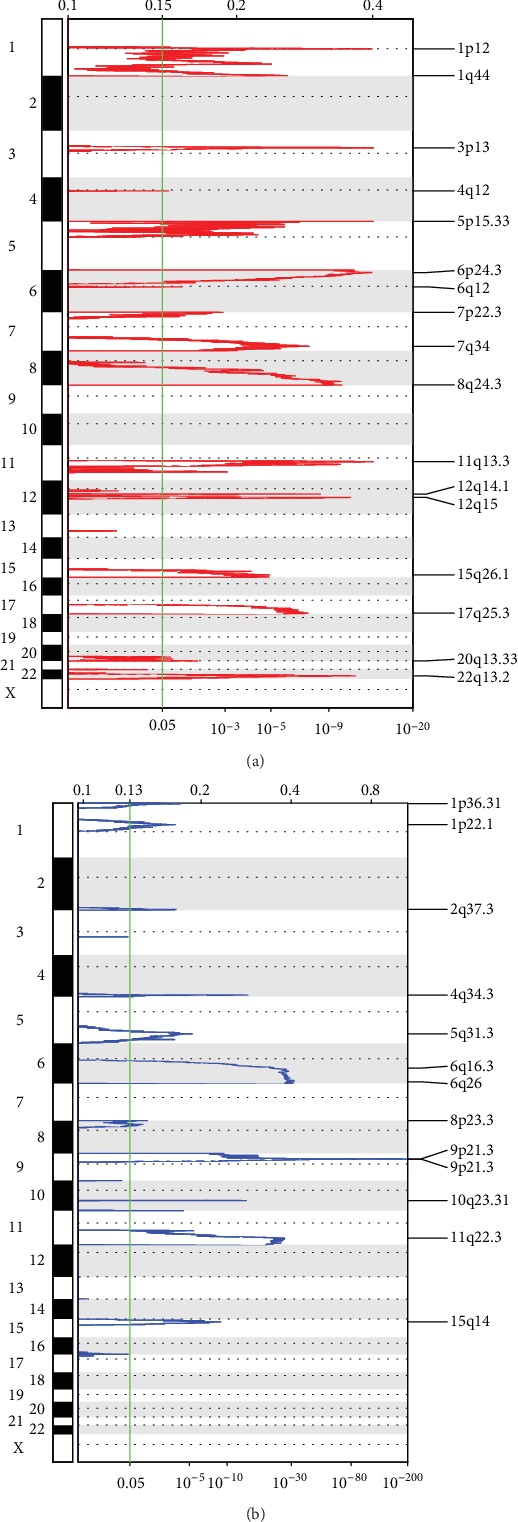
(a) Significantly amplified fragments in the melanoma genome. (b) Significantly deleted fragments in the melanoma genome.

Figure 2(a) shows the results obtained from a significant amplification of the melanoma genome, and Table S2 displays records of genes that were significantly amplified on each fragment. Some examples include *BRAF* which was significantly amplified on the 7q34 segment (*q* value = 3.98E − 08), CCND1 on the 11q13.3 segment (*q* value = 1.46E − 14), and CDK4 on the 12q14.1 segment (*q* value = 5.20E − 09). A total of 1101 genes were amplified.  Figure 2(b) shows a fragment that was notably absent in the melanoma genome. Table S3 contains records of genes that were notably deleted on each fragment, for example, CDKN2A was significantly missing in segment 9p21.3 (*q* value = 1.1011E − 198), PTEN was significantly missing in segment 10q23.31 (*q* value = 1.02E − 14), and SPRED1 was significantly missing in segment 15q14 (*q* value = 1.23E − 09). A total of 1093 genes were missing.

### 3.3. Mutation Data Analysis

**Figure 3:**
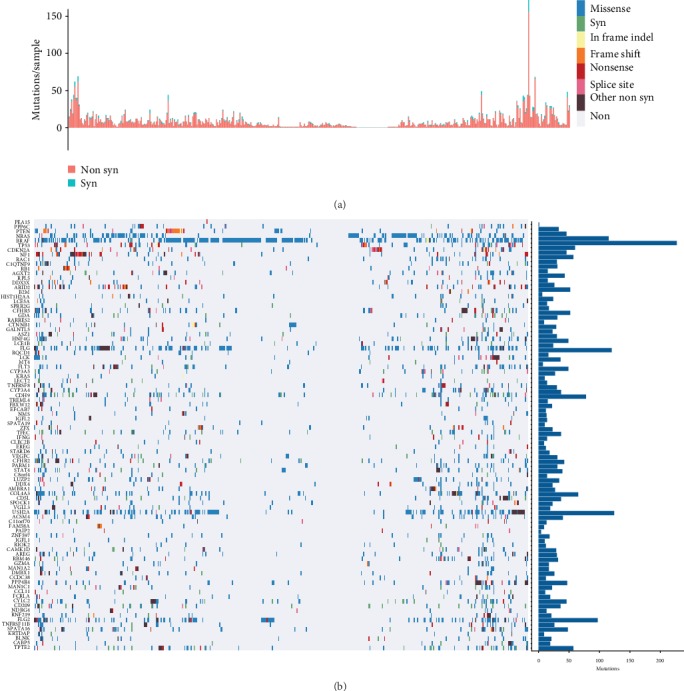
Distribution of 92 genes with significant mutations in melanoma patients. (a) The total number of synonymous and nonsynonymous mutations in 92 genes per patient. (b) The number of samples in which 92 genes were mutated in all samples.

We showed the distribution of synonymous mutations, missense mutations, framework insertions or deletions, framework movements, nonsense mutations, cleavage sites, and other nonsynonymous mutations in the 92 genes distribution within TCGA melanoma patient samples (Figure 3).  Figure 3(a) shows the total number of synonymous and nonsynonymous mutations in 92 genes per patient, and Figure 3(b) shows the number of samples in which 92 genes were mutated. From this analysis, 92 genes were identified, some of which were closely related to the development of cancer, such as *PTEN*, *NRAS*, *BRAF*, *TP53*, and *CDKN2A*.

### 3.4. Pathways and Biological Processes Involved in Copy Number Variant Genes and Mutant Genes

**Figure 4:**
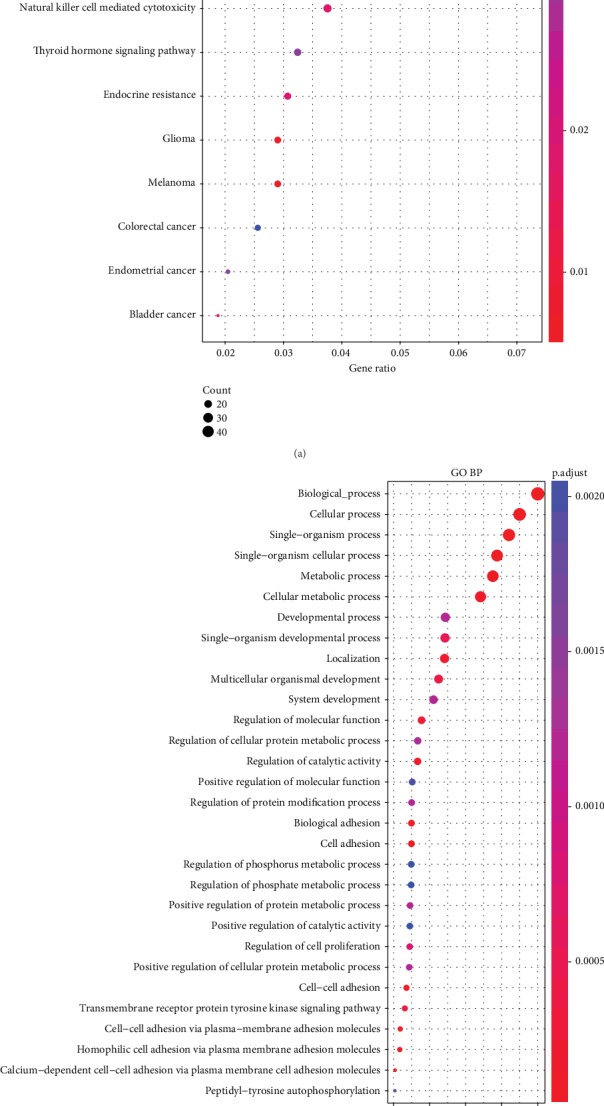
(a) 2286 KEGG pathways involved in genes with copy number variation and mutation. (b) 2286 biological processes involved in the generation of copy number variants and mutations (GO BP).

For amplified and deleted genes recognized by TCGA copy number variation, as well as mutated gene integration, we identified a total of 2286 genes involved in biological processes and pathways. As shown in Figure 4(a), 2286 genes were significantly enriched in melanoma, glioma, and breast cancer. PI3K-Akt signaling pathway and natural killer cell mediated cytotoxicity.  Figure 4(b) displays the 2286 genes which were significantly enriched in biological processes of cancer development, such as system development, regulation of cellular protein metabolic process, regulation of cell proliferation, and cell-cell adhesion.

### 3.5. Random Survival Forests Rank Order of Prognostic Genes

**Figure 5:**
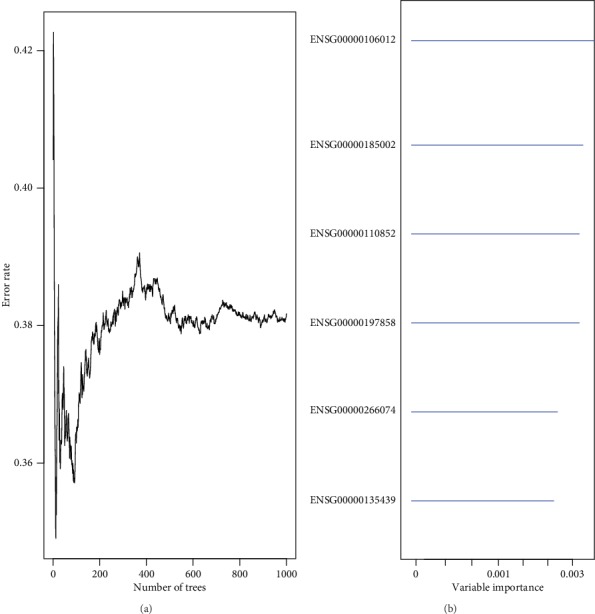
(a) Error rate for the data as a function of trees. (b) Out-of-bag importance values for predictors.

We integrated 2286 genes with copy number amplification, deletion, and mutation and 1569 prognosis-related genes and then selected the intersection of the two groups as candidate genes, which yielded 141 genes. The random survival forest algorithm was used to rank order the prognosis genes (R package random survival forest) using the parameters of *n*_rep_ = 100 and *n*_step_ = 5, representing that the number of Monte Carlo iterations was 100 and the number of preprogressions was 5. Genes with relative importance greater than 0.6 were identified as comprising the final signature.  Figure 5(a) shows the relationship between the error rate and number of classification trees.  Figure 5(b) shows the order of importance of the first six genes out-of-bag.

### 3.6. Creation of a 6-Gene Signature and Division of Samples in the TCGA Training Set

Six genes were identified and subsequently used to construct a prognostic gene signature. For the 6-gene signature identified above, information regarding the importance of HR, *z*-score, and *p* value of the 6 genes and the relative importance is summarized in Table 3.

A multivariate Cox regression analysis method was then used to establish a 6-gene signature. The model used was

(2) $$\begin{array}{c} {\text{Risk}}_{6}=0.2055267*IQCE+0.244203*RFX6+0.1858435*GPAA1+0.2002694*BAHCC1-0.329238*CLEC2B-0.08811327*AGAP2. \end{array}$$

**Figure 6:**
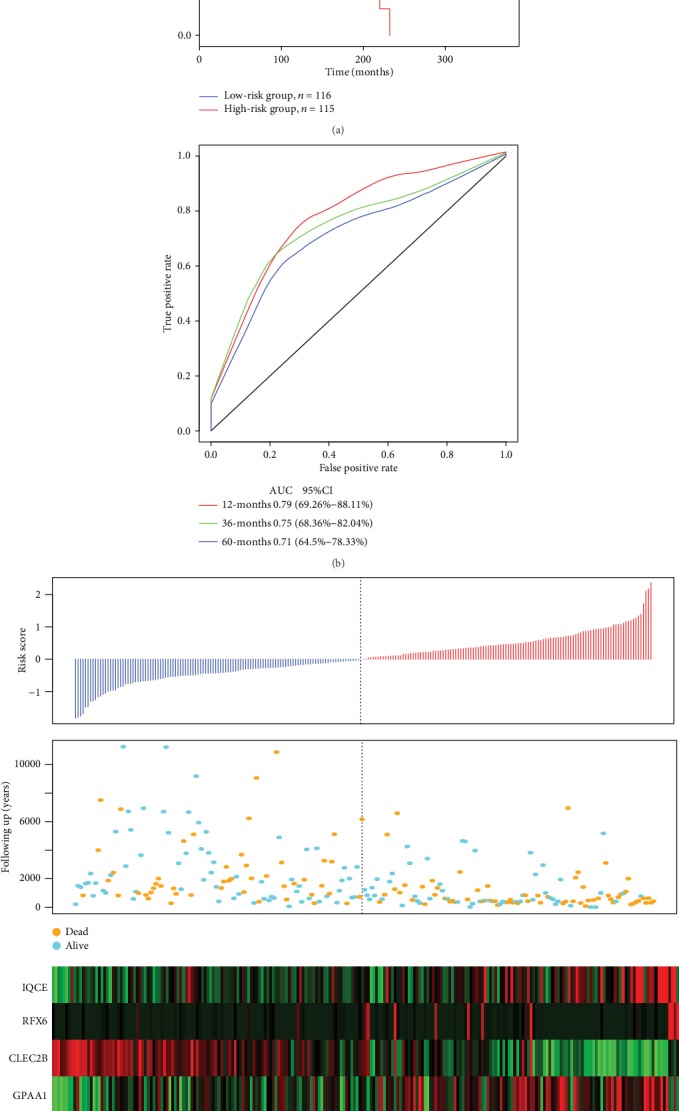
(a) Distribution of the Kaplan-Meier survival curves of the 6-gene signature in the TCGA training set. (b) ROC curves and AUCs of the 6-gene signature classification. (c) Risk score, survival time, survival status, and expression of 6 genes in TCGA training set.

The scoring formula for each sample was the sum of the above gene expression values∗coefficients, with the sample scoring median of -0.03765742 selected as a cutoff to divide the samples into high-risk group and low-risk group.  Figure 5 shows the classification effect in the TCGA training set. 116 patients were classified within the low-risk group and 115 patients in the high-risk group (Figure 6(a)). Differences between the two groups were statistically significant (log − rank *p* = 6.861462E − 08).  Figure 6(b) shows the ROC curves with AUCs of 0.79, 0.75, and 0.71 for one, three, and five years, respectively.  Figure 6(c) shows that as the patient's risk score increased, their survival time significantly decreased, with this effect being more prevalent in the high-risk group.

As the risk value increased, the expression levels of the six different signature genes changed. According to the above results, high expression levels of *IQCE*, *RFX6*, *GPAA1*, and *BAHCC1* were associated with increase in risk factors, while the high expression levels of *CLEC2B* and *AGAP2* were related to protection factors.

### 3.7. Detection of Robustness of 6-Gene Signature in the TCGA Test Set

**Figure 7:**
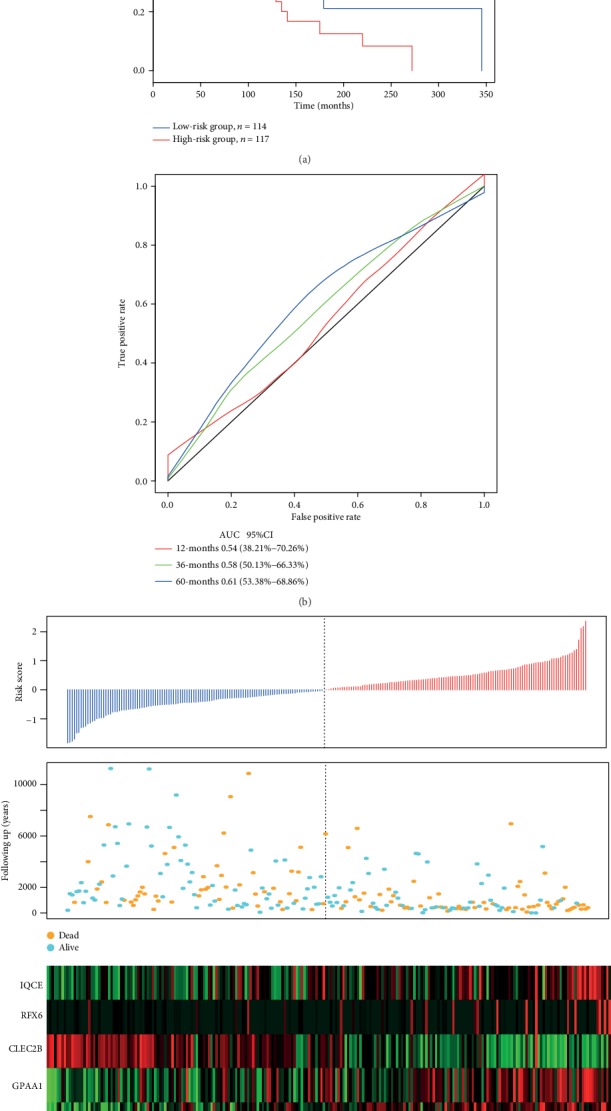
(a) 6-gene signature Kapan-Meier survival curve distributions in the TCGA test set. (b) ROC curves and AUCs of the 6-gene signature classification. (c) TCGA test set focused on risk score, survival time, survival status, and the expression of six genes.

Figure 7 contains an illustration of classification results in the TCGA test set. There were 114 patients classified as low-risk and 117 comprising the high-risk group (Figure 7(a)), with differences between these groups achieving statistical significance (log − rank *p* = 0.029). AUC values from the ROC curves were 0.54, 0.58, and 0.61 for one, three, and five years, respectively (Figure 7(b)).  Figure 7(c) shows that similar results obtained from the TCGA training set. As the risk value increased, survival time significantly decreased, with this effect being more predominant in the high-risk group. As the risk value increased, the expression levels of the six different signature genes changed. High expression levels of *IQCE*, *RFX6*, *GPAA1*, and *BAHCC1* were associated with high risk factors, while high expression levels of *CLEC2B* and *AGAP2* indicated low risk and served as protective factors.

**Figure 8:**
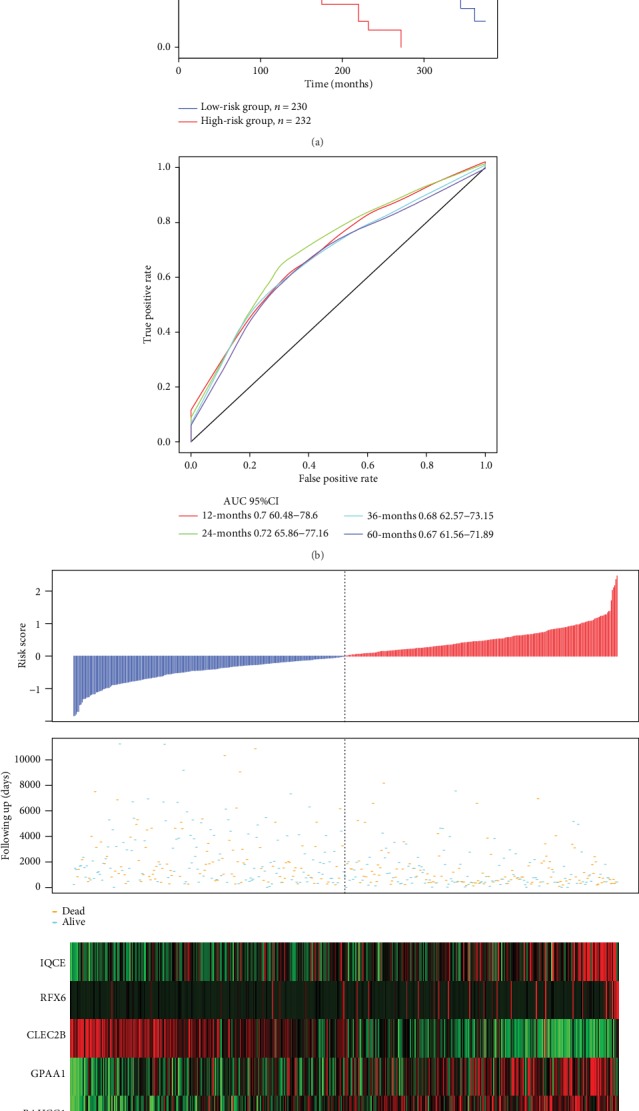
(a) 6-gene signature KM survival curve distributions in the TCGA dataset. (b) ROC curves and AUCs of the 6-gene signature classification. (c) Risk scores in the TCGA dataset, survival time, survival status, and expression of six genes.

We also assessed the robustness of the model (the training set and test set) in all samples. In this assessment, the same model as that of the TCGA training set and the same cutoff was used for verification in all TCGA datasets. The classification effect in the TCGA test set is presented in Figure 6. From this analysis, 230 patients were classified as low-risk and 232 as high-risk patients (Figure 8(a)), with differences between these two groups being statistically significant (log − rank *p* < 0.001). AUC values from ROC curves were 0.7, 0.72, 0.68, and 0.67 for one, two, three, and five years, respectively (Figure 8(b)). Similar results were obtained from the TCGA training set (Figure 8(c)). As the risk value increased, the survival time significantly decreased, with this effect being more predominant in the high-risk group. Moreover, increased expression of the six different signature genes was observed as the risk value increased. High expression levels of *IQCE*, *RFX6*, *GPAA1*, and *BAHCC1* were associated with high-risk factors, while high expression levels of *CLEC2B* and *AGAP2* with low risk and served as protective factors.

### 3.8. Verification of 6-Gene Signature Robustness in the External Independent Dataset GSE19234 and GSE65904

**Figure 9:**
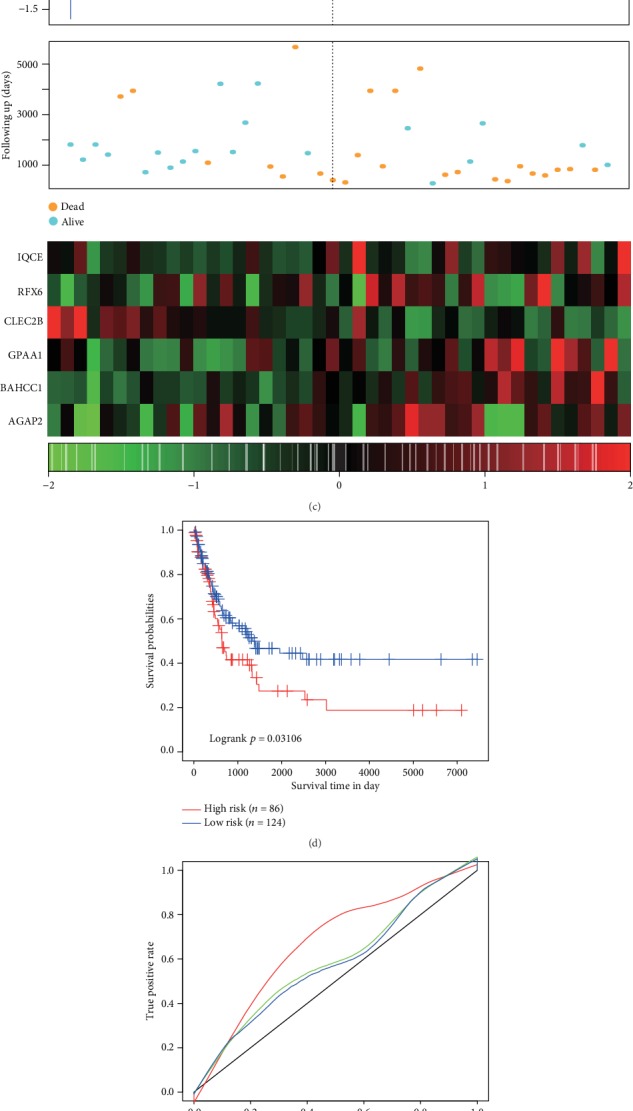
(a) 6-gene signature KM survival curve distributions in GSE19234. (b) ROC curves and AUCs of the 6-gene signature classification in GSE19234. (c) Risk score in GSE19234, survival time, survival status, and expression of 6 genes. (d) 6-gene signature KM survival curve distributions in GSE65904. (e) ROC curves and AUCs of the 6-gene signature classification in GSE65904. (f) Risk score in GSE65904, survival time, survival status, and expression of 6 genes.

The classification effect in GSE19234 and GSE65904 is shown in Figure 9. 18 patients were classified as low risk and 26 patients as high risk (Figure 9(a)), with differences between these two groups being statistically significant (log − rank *p* = 0.013). AUC values from ROC curves were 0.54, 0.84, and 0.79 for one, three, and five years, respectively (Figure 9(b)). Similar results were obtained from the TCGA training set (Figure 9(c)). 124 patients were classified as low risk and 86 patients as high risk (Figure 9(d)), with differences between these two groups being statistically significant (log − rank *p* = 0.031). AUC values from ROC curves were 0.68, 0.59, and 0.59 for one, two, and three years, respectively (Figure 9(e)). Similar results were obtained from the TCGA training set (Figure 9(f)). As the risk value increased, survival time significantly decreased, with this effect being more predominant in the high-risk group. Moreover, increased expression of the six different signature genes was observed as the risk value increased. High expression levels of *IQCE*, *RFX6*, *GPAA1*, and *BAHCC1* were associated with high risk factors, while high expression levels of *CLEC2B* and *AGAP2* with low risk and served as protective factors.

### 3.9. Analysis of Clinical Independence of the 6-Gene Signature Model

We systematically analyzed the clinical information of TCGA, GSE19234, and GSE65904 patient records, including age, gender, pathology T phase, N phase, M phase, tumor stage, and our 6-gene signature grouping information as shown in Table 4.

In the TCGA training set, the univariate Cox proportional hazard regression analysis revealed that the high-risk group, age, pathologic T3, pathologic T4, pathologic N2, pathologic N3, and tumor stage III/IV were all significantly associated with survival. However, when applying the corresponding multivariate Cox regression analysis, we found that only the high-risk group (HR = 1.87, 95%CI = 1.11 − 3.14, *p* = 0.018), age (HR = 1.02, 95%CI = 1.001 − 1.03, *p* = 0.037), pathologic T4 (HR = 11.88, 95%CI = 3.31 − 42.52, *p* = 1.43E − 04), and tumor stage II (HR = 0.28, 95%CI = 0.092 − 0.82, *p* = 0.022) were clinically independent.

In the TCGA test set, the univariate Cox proportional hazard regression analysis revealed that the high-risk group, age, pathologic T4, pathologic N3, pathologic M1, and tumor stage III/IV were all significantly associated with survival. However, we found that only pathologic N3 (HR = 5.86, 95%CI = 1.4 − 24.53, *p* = 0.0154) was clinically independent from corresponding multivariate Cox regression analysis. The high-risk group showed a similar trend, but this effect failed to achieve statistical significance (HR = 1.41, 95%CI = 0.86 − 2.29, *p* = 0.166).

In GSE19234, the univariate Cox proportional hazard regression analysis revealed that the high-risk group and tumor stage IV was significantly associated with survival. The corresponding multivariate Cox regression analysis indicated that the high-risk group (HR = 3.34, 95%CI = 1.2 − 9.3, *p* = 0.021) and tumor stage IV (HR = 4.2915, 95%CI = 1.46 − 12.56, *p* = 0.008) were clinically independent.

In GSE65904, the univariate Cox proportional hazard regression analysis revealed that the high-risk group was significantly associated with survival. The corresponding multivariate Cox regression analysis indicated that the high-risk group (HR = 1.492, 95%CI = 1.002 − 2.220, *p* = 0.049) was clinically independent.

Taken together, the above results indicated that the 6-gene signature model can serve as a prognostic indicator independent of other clinical factors and contains an independent predictive capacity of value for clinical application.

**Figure 10:**
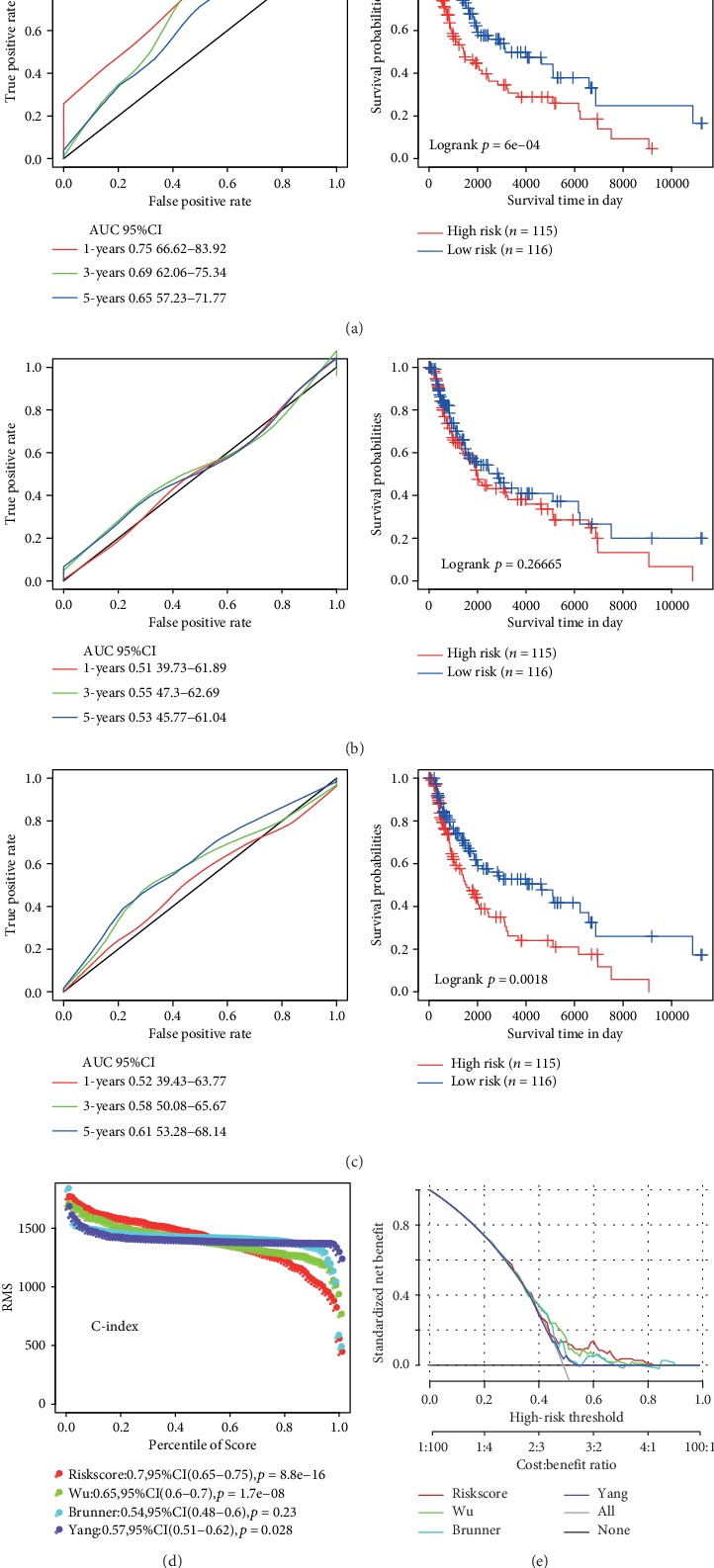
Comparison and analysis between the 6-gene signature model and other existing models. (a) AUC and KM curve of Wu's model. (b) AUC and KM curve of Brunner's model. (c) AUC and KM curve of Yang's model. (d) Three models and the RMS curve of the 6-gene signature. (e) DCA curve of the RMS curve of the three models and the 6-gene signature.

In addition, we compared some other known models, by studying three recently published lung cancer prognosis model, such as Wu et al. [15], Brunner et al. [16], and Yang et al. [17]. In order to make the model comparable, we carried out the following work. According to the corresponding gene in the three models, we used the same method to calculate the risk score of each sample in the TCGA and assessed the ROC of each model. In addition, we divided the sample into high-risk group and low-risk group according to the median risk score, and then we calculated the OS prognosis between the two groups. We found that the overall performance of our model was better than that of the above three models, as shown in Figures 10(a)–10(c). The restricted mean survival curves of these models were also compared, as shown in Figure 10(d), from which it can be seen that our model has the highest c-index among the four models. That means that our model has an advantage in long-term survival prediction. Meanwhile, we compared the prediction effect of the 6-gene signature with that of the three models through DCA curves, and the results showed that the performance of our model is better than that of the other three models as indicated in Figure 10(e).

### 3.10. Use of GSEA to Analyze Pathways Enriched in High-Risk Group and Low-Risk Group

**Figure 11:**
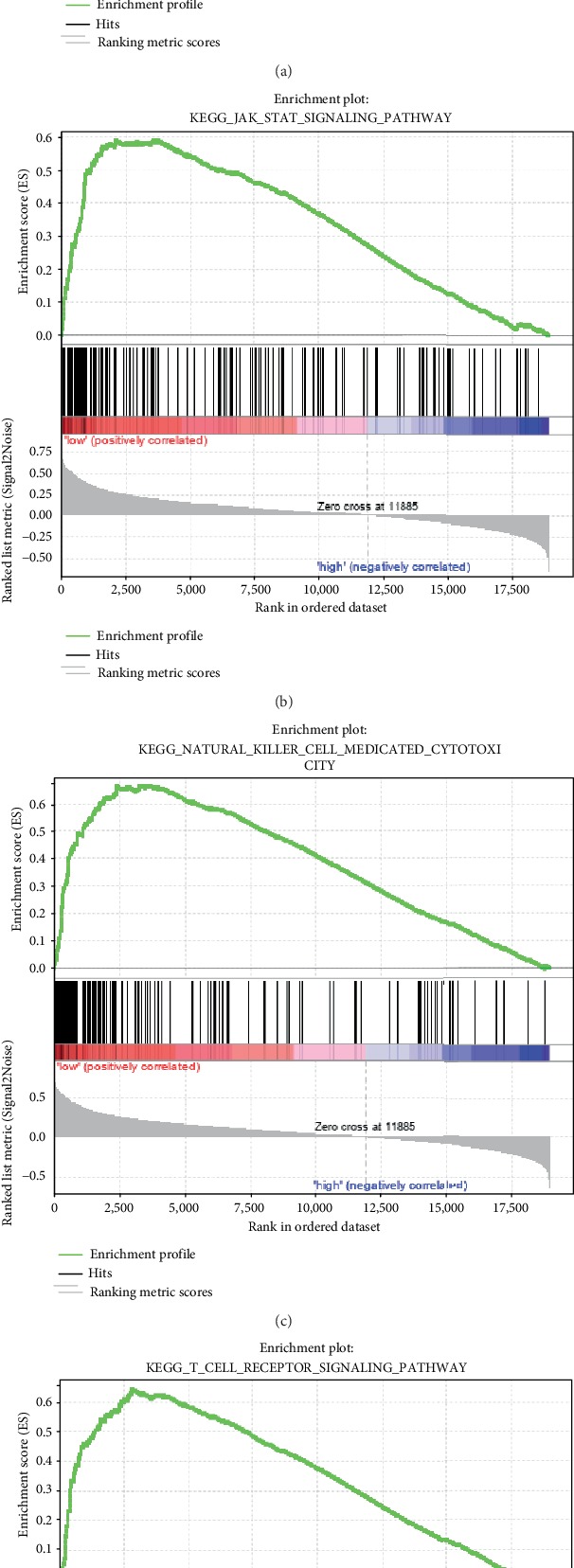
Enriched pathways in the high-risk group and low-risk group as obtained in the 6-gene signature.

We obtained a significantly enriched path as indicated in Table S4. Some examples of significantly enriched pathways were presented in Figure 11, including cell adhesion molecules cams, JAK-STAT signaling pathway, natural killer cell-mediated cytotoxicity, and T cell receptor signaling pathway. All pathways were significantly related to the development and metastasis of melanoma.

### 3.11. Experimental Verification of the Biomarker Screening Results with qRT-PCR and Immunohistochemistry Analysis

**Figure 12:**
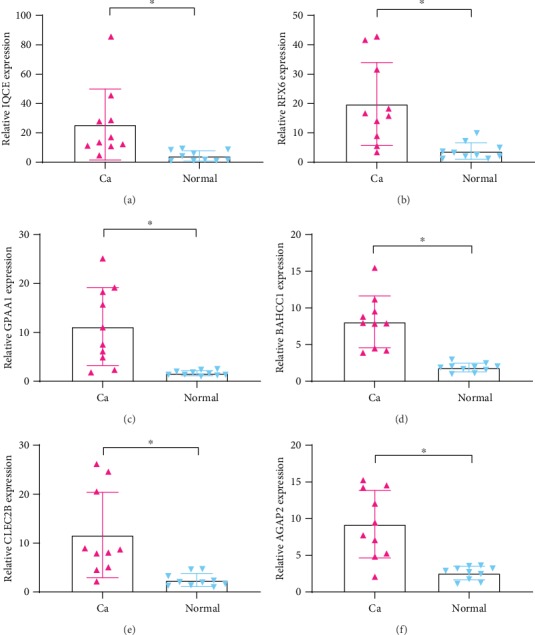
The expression of *IQCE*, *RFX6*, *GPAA1*, *BAHCC1*, *CLEC2B*, and *AGAP2* significantly increased in melanoma tissues compared to normal tissues (^∗^*p* < 0.01, *n* = 10). Ca: melanoma tissues; Normal: normal healthy tissues.

**Figure 13:**
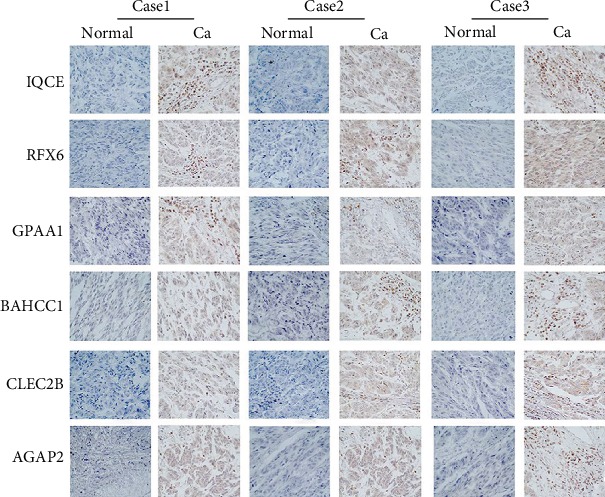
The *IQCE*, *RFX6*, *GPAA1*, *BAHCC1*, *CLEC2B*, and *AGAP2* were highly expressed in melanoma tumor tissues (Ca: brown) in comparison with the normal tissues (Normal: blue). Ca: melanoma tissues; Normal: normal healthy tissues.

In order to verify whether *IQCE*, *RFX6*, *GPAA1*, *BAHCC1*, *CLEC2B*, and *AGAP2* were highly expressed in melanoma tissues as predicted, we experimentally confirmed this by qRT-PCR and immunohistochemical staining using melanoma tissues extracted from 10 patients. The qRT-PCR result is shown in Figure 12; *IQCE*, *RFX6*, *GPAA1*, *BAHCC1*, *CLEC2B*, and *AGAP2* were all highly expressed in melanoma tissues compared with normal healthy control (*p* < 0.05, Student's *t*-test, *n* = 10). In addition, immunohistochemistry analysis demonstrated that *IQCE*, *RFX6*, *GPAA1*, *BAHCC1*, *CLEC2B*, and *AGAP2* were highly expressed in melanoma tissues compared with normal tissue (Figure 13).

## 4. Discussion

Melanoma leads to 90% of mortalities among all skin cancers [18]. The incidence of melanoma in people over 60 years of age has risen sharply, especially in Europe, and the incidence of melanoma continues to increase [19]. Melanoma is a genetically heterogeneous disorder, and the lesions located in different anatomical locations exhibit different molecular features. The latest research progress in melanoma provides possible method for discrete classifications of melanoma that would consider not only the epidemiology and pathology but also the mutational profiles and other novel biomarkers [20]. Melanoma is highly heterogeneous in terms of prognosis as melanoma patients with same TNM stages show different survival time. Moreover, as melanoma is increasingly detected and treated in the early stages, traditional clinicopathological indicators, such as tumor size, vascular invasion, portal vein thrombosis, and TNM staging have become less effective in predicting individual outcomes. This is especially true for risk stratification, as no “one size fits all” treatment strategy has been proven to be effective [21]. It is clear that screening for prognostic molecular markers that fully reflect the biological characteristics of tumors is critical for individualized prevention and treatment of melanoma patients. In this study, we analyzed the expression profiles of 716 melanoma samples from TCGA and GEO databases as associated with OS. Based on this analysis, a related robust 6-gene signature which is independent of clinical factors was generated and verified.

We assessed the effectiveness of this 6-gene signature using multiomics data, including transcriptome, copy number variation data, and mutation data, to identify disease-associated genes. Based on a TCGA dataset containing 462 samples, a potential prognostic six-marker lncRNA was identified. The signature included *IQCE*, *RFX6*, *GPAA1*, *BAHCC1*, *CLEC2B*, and *AGAP2*. Among these lncRNAs, high expression levels of *IQCE*, *RFX6*, *GPAA*1, and *BAHCC1* were associated with risk factors. High expression levels of *CLEC2B* and *AGAP2* were found to be protective factors. According to the previous reports, *RFX6* can be used as a marker of prostate cancer [22, 23]. *GAPP1* is closely related to prognosis in gastric cancer, head and neck squamous carcinoma, and hepatocellular carcinoma [24–26]. *BAHCC1* is closely related to prognosis in hepatocellular carcinoma [27]. *CLEC2B* is a marker of clear cell renal cell carcinoma and other tumors [28–31]. *AGAP2* is closely related to prognosis in gastric cancer and prostate cancer [32, 33]. *IQCE* has not been previously reported to be related to cancer. Ours is the first study to suggest that it can be used as new prognostic markers of melanoma.

The 6-gene signature of multiomics data recognition is robust and can achieve stable prediction performance in datasets of different platforms. We systematically analyzed the patient record and clinical information in TCGA, GSE19234, and GSE65904 datasets, including age, gender, pathology, T stage, N stage, M stage, tumor stage, and our 6-gene signature group information. Univariate Cox regression analysis and multifactor Cox regression analysis showed that our multiple omics data of 6-gene signature has strong clinical independence and can maintain stable under the influence of multiple clinical factors.

The classification ability of this characteristic lncRNA was verified on a TCGA test dataset and GSE19234 and GSE65904 datasets. Subsequent analysis supported the conclusion that this 6-lncRNA feature showed reliable prediction accuracy. The enriched pathways in the high-risk group and low-risk group obtained by GSEA analysis of 6-gene signature were significantly related to the occurrence and development of melanoma, suggesting its potential as a prognostic marker for clinical diagnosis. For example, cell adhesion molecules cams, JAK-STAT signaling pathway, natural killer cell-mediated cytotoxicity, and T cell receptor signaling pathway were all related to a variety of tumors [34–37]. To the best of our knowledge, the prognostic value of this multimarker feature in melanoma has not previously been reported. Therefore, our findings provide new insights into improving risk stratification and survival prediction for melanoma patients.

Although we identified potential candidate genes for melanoma prognosis in large samples through bioinformatic techniques, some limitations of this study should be noted. First, the sample lacks some clinical follow-up information. For example, we did not consider factors such as the presence of other health conditions within these patients to distinguish the prognostic biomarkers. Second, although we have carried out experimental verification, our sample size was not large enough. Therefore, further genetic and experimental studies involving larger sample size and experimental validation are required.

## 5. Conclusions

In summary, we constructed a 6-gene signature (*IQCE*, *RFX6*, *GPAA1*, *BAHCC1*, *CLEC2B*, and *AGAP2*) as a novel prognostic marker with respectable AUCs in both the training and validation sets in this study, which was independent of clinical features. Compared with clinical features, this gene classifier can improve survival risk prediction. Therefore, we recommend using this classifier to assess the prognostic risk of melanoma.

## Figures and Tables

**Table 1:** Clinical information of the four datasets.

Characteristic		TCGA training datasets (*n* = 231)	TCGA test datasets (*n* = 231)	GSE19234 (*n* = 44)	GSE65904 (*n* = 210)
Age (years)	≤50	153	64	13	40
	>50	78	167	31	169

Survival status	Living	118	124	20	108
	Dead	113	107	24	102

Gender	Female	96	78	16	86
	Male	35	153	28	124

pathologic_T	T1	23	18		
	T2	40	37		
	T3	45	45		
	T4	70	82		

pathologic_N	N0	114	115		
	N1	36	37		
	N2	27	22		
	N3	31	25		

pathologic_M	M0	205	206		
	M1	17	7		

Tumor stage	Stage I	42	35		
	Stage II	66	73		
	Stage III	83	87	39	
	Stage IV	16	7	5	

**Table 2:** Upper 20 prognosis-related gene information.

ENSG ID	HR	Coefficient	*z*-score	*p* value
ENSG00000167491	1.624708014	0.485328116	5.223401669	1.76E-07
ENSG00000110852	0.602961562	-0.505901829	-4.998288473	5.78E-07
ENSG00000101542	1.405581206	0.340450887	4.841619291	1.29E-06
ENSG00000070081	0.604807853	-0.50284447	-4.776054928	1.79E-06
ENSG00000106560	0.618291849	-0.480794685	-4.653630952	3.26E-06
ENSG00000123609	0.636638101	-0.451553915	-4.652856943	3.27E-06
ENSG00000239713	0.61079206	-0.492998705	-4.634911328	3.57E-06
ENSG00000162645	0.650961966	-0.429304062	-4.578510783	4.68E-06
ENSG00000013392	0.620058745	-0.477941055	-4.565733982	4.98E-06
ENSG00000182179	0.664881034	-0.408147151	-4.535593838	5.74E-06
ENSG00000162654	0.646580192	-0.436058048	-4.517325609	6.26E-06
ENSG00000104848	1.384886188	0.325617961	4.51248348	6.41E-06
ENSG00000168404	0.635103649	-0.453967066	-4.472065487	7.75E-06
ENSG00000163001	0.653569834	-0.42530589	-4.419121401	9.91E-06
ENSG00000151500	0.640197346	-0.445978797	-4.391908428	1.12E-05
ENSG00000117151	0.644975676	-0.438542675	-4.386964355	1.15E-05
ENSG00000156587	0.65353037	-0.425366275	-4.363773665	1.28E-05
ENSG00000132274	0.638251257	-0.449023254	-4.350642875	1.36E-05
ENSG00000080603	1.494851402	0.402026805	4.34413171	1.40E-05
ENSG00000177409	0.664707433	-0.408408286	-4.337688225	1.44E-05

**Table 3:** The 6 genes significantly associated with the overall survival in training set patients.

Ensemble gene ID	Symbol	HR	*z*-score	*p* value	Importance	Relative importance
ENSG00000106012	IQCE	1.34	3.462151	5.36E-04	0.0034	0.94
ENSG00000185002	RFX6	1.23	2.977574	2.91E-03	0.0034	0.92
ENSG00000110852	CLEC2B	0.6	-4.998288	5.78E-07	0.0034	0.92
ENSG00000197858	GPAA1	1.32	2.681788	7.32E-03	0.0029	0.8
ENSG00000266074	BAHCC1	1.39	3.46767	5.25E-04	0.0029	0.78
ENSG00000135439	AGAP2	0.72	-2.741749	6.11E-03	0.0024	0.66

**Table 4:** Identification of prognostic-related clinical factors and clinical independence using univariate and multivariate Cox regression analyses in the TCGA training set, TCGA test set, GSE19234, and GSE65904.

Variables	Univariate analysis			Multivariable analysis		
	HR	95% CI of HR	*p* value	HR	95% CI of HR	*p* value
TCGA training datasets						
6-gene risk score						
Low-risk group	1 (reference)			1 (reference)		
High-risk group	2.81	1.91-4.14	1.63E-07	1.87	1.11-3.14	0.018
Age	1.03	1.01-1.04	1.76E-05	1.02	1.001-1.03	0.03736
Gender female	1 (reference)					
Gender male	1.14	0.77-1.67	0.51	0.98	0.61-1.58	0.941573
Pathologic T1	1 (reference)			1 (reference)		
Pathologic T2	2.24	0.90-5.53	0.08	1.88	0.68-5.21	0.227
Pathologic T3	2.99	1.22-7.3	0.02	5.52	1.50-20.24	0.01
Pathologic T4	5.75	2.36-13.96	1.10E-04	11.88	3.31-42.52	1.43E-04
Pathologic N0	1 (reference)			1 (reference)		
Pathologic N1	1.49	0.85-2.61	0.163	2.58	0.31-20.90	0.374
Pathologic N2	1.94	1.06-3.55	0.03	2.6	0.31-21.56	0.375
Pathologic N3	2.68	1.53-4.67	5.30E-04	7.35	0.88-60.98	0.065
Pathologic M0	1 (reference)			1 (reference)		
Pathologic M1	1.66	0.83-3.28	0.151	1.26	0.42-3.74	0.67
Tumor stage I	1 (reference)			1 (reference)		
Tumor stage II	1.64	0.92-2.89	0.089	0.28	0.092-0.82	0.022
Tumor stage III/IV	2.25	1.32-3.79	0.002	0.22	0.024-2.0024	0.18
Validation cohort, TCGA test datasets, GSE19234, and GSE65904						
TCGA test datasets						
6-gene risk score						
Low-risk group	1 (reference)			1 (reference)		
High-risk group	1.54	1.04-2.26	0.029	1.41	0.86-2.29	0.166
Age	1.02	1.007-1.034	0.003	1.01	0.99-1.02	0.223
Gender female	1 (reference)			1 (reference)		
Gender male	1.28	0.83-1.97	0.26	1.23	0.70-2.16	0.454
Pathologic T1	1 (reference)			1 (reference)		
Pathologic T2	0.98	0.42-2.26	0.957	1.35	0.48-3.75	0.563
Pathologic T3	1.4	0.62-3.12	0.409	1.25	0.33-4.69	0.737
Pathologic T4	2.47	1.13-5.36	0.023	2.3	0.62-8.45	0.21
Pathologic N0	1 (reference)			1 (reference)		
Pathologic N1	1.46	0.85-2.49	0.168	2.12	0.57-7.94	0.261
Pathologic N2	1.11	0.54-2.24	0.78	2.29	0.57-9.12	0.237
Pathologic N3	3	1.47-6.09	0.002	5.86	1.4-24.53	0.015
Pathologic M0	1 (reference)			1 (reference)		
Pathologic M1	4.35	1.01-18.62	0.047	5.307	0.54-51.81	0.151
Tumor stage I	1 (reference)			1 (reference)		
Tumor stage II	1.51	0.83-2.73	0.172	1.14	0.37-3.5	0.813
Tumor stage III/IV	1.86	1.06-3.28	0.03	0.88	0.18-4.21	0.882
GSE19234						
6-gene risk score						
Low-risk group	1 (reference)			1 (reference)		
High-risk group	3.22	1.18-8.76	0.022	3.34	1.2-9.3	0.021
Age	1.01	0.98-1.02	0.579	1	0.97-1.023	1
Gender female	1 (reference)			1 (reference)		
Gender male	0.76	0.33-1.75	0.526	0.93	0.4-2.15	0.868
Tumor stage III	1 (reference)			1 (reference)		
Tumor stage IV	4.03	1.43-11.38	0.008	4.29	1.46-12.56	0.008
GSE65904						
6-gene risk score						
Low-risk group	1 (reference)			1 (reference)		
High-risk group	1.53	1.036-2.265	0.032	1.492	1.002-2.220	0.0488
Age	0.998	0.984-1.012	0.796	1.001	0.987-1.015	0.927
Gender female	1 (reference)			1 (reference)		
Gender male	0.748	0.496-1.13	0.169	0.788	0.522-1.192	0.259

## Data Availability

The data used to support the findings of this study are available from the corresponding author upon request.

## Supplementary Materials

Supplementary 1 Table S1: the 1569 candidate prognostic genes, coefficients, *z*-score, *p* values, and HR.

Supplementary 2 Table S2: records of genes that are significantly amplified on each fragment.

Supplementary 3 Table S3: records of genes that are notably deleted on each fragment.

Supplementary 4 Table S4: all significantly enriched path.
